# Sex and spouse conditions influence symptoms of anxiety, depression, and posttraumatic stress disorder in both patients admitted to intensive care units and their spouses

**DOI:** 10.5935/0103-507X.20180004

**Published:** 2018

**Authors:** Henrique Souza Barros de Oliveira, Renata Rego Lins Fumis

**Affiliations:** 1 Intensive Care Unit, Hospital Sírio-Libanês - São Paulo (SP), Brazil.

**Keywords:** Anxiety, Depression, Spouses, Stress disorders, post-traumatic, Intensive care units, Female

## Abstract

**Objectives:**

To assess the effect of sex and spouse condition on symptoms of anxiety, depression and posttraumatic stress symptoms in patients and their spouses.

**Methods:**

A prospective study conducted in a 22-bed adult mixed intensive care unit in a tertiary hospital in São Paulo, Brazil. Patients and their spouses were enrolled 2 days after intensive care unit admission. They were interviewed while in the intensive care unit using the Hospital Anxiety and Depression Scale. At 30 and 90 days after intensive care unit discharge, they completed the Impact of Event Scale and Hospital Anxiety and Depression Scale by phone.

**Results:**

From March 2011 to March 2013, we analyzed 118 patients and their spouses. We observed that female sex was associated with higher scores than male sex was in terms of the anxiety Hospital Anxiety and Depression Scale - subscale (p = 0.032) and depression (p = 0.034). There was no association between sex and posttraumatic stress disorder symptoms. However, spouses had higher Impact of Event Scale points compared with patients (p = 0.001).

**Conclusions:**

Female sex was associated with anxiety and depression, and spouses were more vulnerable to post-traumatic stress symptoms than the patients were. Increasing age and a later time of assessment were also associated with lower scores on the Impact of Event Scale.

## INTRODUCTION

Admission to an intensive care unit (ICU) exposes the patient to serious stressors, such as distress related to the disease, being in pain, having tubes in the nose and/or mouth, loss of control, sleep deprivation, physical limitation, and not being able to communicate.^(1-3)^ Furthermore, patients and family members commonly experience high rates of anxiety and depression symptoms as well as posttraumatic stress disorder (PTSD) both during and after ICU discharge.^(4-8)^ Previous studies highlighted that family members of ICU-admitted patients, particularly their spouses, suffer relevant psychological distress, occasionally even more than their beloved ones; therefore, the family members need support both during the ICU stay and during the follow-up period.^(8)^ Importantly, follow-up studies have suggested that patients and their family members may experience different levels of anxiety, depression and post-traumatic distress in various ICU recovery periods.^(9,10)^

Some factors may contribute to increased incidence of these symptoms, including female sex, spouses, young age, need for mechanical ventilation, and severity of disease.^(6-10)^ Although the literature has identified some risk factors for anxiety, depression and PTSD in critically ill patients and their family members, few studies have assessed the effect of female gender on the development of these symptoms in a specific population, such as patients admitted to an intensive care unit and their spouses. Girard et al. observed that high levels of PTSD symptoms after critical illness requiring mechanical ventilation were most likely to occur in female patients. According to the authors, it is of the utmost importance to understand the risk factors that may facilitate preventive strategies and to direct screening for symptoms of PTSD after critical illness.^(11)^

The aim of this study was to assess the effect of sex and spouse conditions on the symptoms of anxiety, depression and posttraumatic stress symptoms in patients and their spouses during ICU stays and at 30 and 90 days following ICU discharge.

## METHODS

This is a secondary analysis of a prospective study conducted in a tertiary private teaching hospital, Hospital Sírio-Libanês, in São Paulo, Brazil.^(8)^ The institutional review board (IRB), called the "*Comitê de Ética em Pesquisa da Sociedade Beneficente de Senhoras do Hospital Sírio Libanês*", reviewed and approved this study (HSL - protocol number 2010/44). All patients with more than 48 hours of ICU stay and their spouses were invited to participate and sign written informed consent. The study was conducted in a medical-surgical unit that contains 22 private rooms. A family member can stay with the patient 24 hours per day in their private room (for one patient only) and rotate with another visitor at any time during the day or night. They are allowed to sleep in the patient's room (which contains a sofa bed or a comfortable armchair, bathroom and TV). In addition, the ICU has two visitation periods, in which up to two visitors are allowed in the patient's room at the same time. During invasive procedures (intubation, catheterization), family members are invited to remain outside the room.

The professional-to-bed ratios in the ICU are as follows: nurse 1:4; nurse-assistant 1:2; physician 1:6 (dayshift) and 1:10 (night shift). This ICU has a 24-hour visitation policy, and family members remain with the patient for an average of 12 hours per day.^(8)^

The inclusion criteria for patients were age over 18 years and more than 48 hours of ICU stay. The exclusion criteria for patients were psychiatric disorders, severe neurologic disease, illness too severe to answer at the first assessment or any difficulty with follow-up due to physical impairment or limitations (such as hearing, incapacity to speak, language barriers, or being too old to participate, e.g., if advanced age causes limitation in reading, understanding or speaking on the phone). We also invited their spouses (wife, husband or their partners) if they were involved with the patient's care (visiting the patient at least two days). The exclusion criteria for spouses included psychiatric disorders. For patients and spouses, psychiatric disorders included anxiety and depression under drug treatment at ICU admission. After 48 hours of ICU stay, both patient and their spouse were approached. If the patient was unable to participate at first assessment because of clinical conditions (e.g., mechanical ventilation, delirium), they were excluded. During the ICU stay, when patients were able to participate, they were assessed and interviewed. At 30 and 90 days, only those who participated in the ICU period were followed up.

Patients and their spouses were interviewed while in the ICU using the Hospital Anxiety and Depression Scale (HADS). At 30 and 90 days after ICU discharge, they were also interviewed by phone to complete the Impact of Event Scale (IES) and HADS. The HADS score for each subscale (anxiety and depression) ranges from 0 - 21, and a cut-off of > 10 was used to depict each condition. The scores for the entire scale (emotional distress) range from 0 - 42, with higher scores indicating more distress. The IES score has been used for many years and seems reliable across a broad range of traumatic events, and it can be easily applied during a telephone interview. The IES is not a tool for diagnosing PTSD, but it does detect symptoms indicating a risk of PTSD. It includes 15 items, seven of which measure intrusive symptoms (for example, intrusive thoughts, nightmares, intrusive feelings and imagery) and eight of which measure avoidance symptoms (numbing of responsiveness, avoidance of feelings, situations, and ideas). Respondents are asked to rate the items according to how often each has occurred in the past 7 days. The IES score ranges from 0 - 75 points, with higher scores indicating more severe post-traumatic stress symptoms. Patients were classified as having low or high IES scores, using 30 as the cutoff, in agreement with previous reports that higher than 30 points indicates post-traumatic stress reaction with a significant risk of PTSD. To ensure optimal quality of the data, all interviews were conducted by the same person, a psychologist with ICU interview experience. Both scales were previously validated in Brazil.^(12,13)^

For each patient, the following information was collected: age, sex, level of education, cause of ICU admission, cancer disease, Simplified Acute Physiology Score (SAPS) 3, Glasgow Coma Scale, Sequential Organ Failure Assessment (SOFA), ICU length of stay, need of mechanical ventilation, renal replacement therapy in the ICU, delirium (positive Confusion Assessment Method for the Intensive Care Unit - CAM-ICU), and final outcome in the ICU. The following information was supplied by the spouse: sex, age, level of education, religious belief, previous ICU experience and how much time per day is spent with the patient in the ICU.

### Statistical analysis

We performed descriptive analysis of continuous variables using the mean and standard deviation or median and interquartile range and frequencies for categorical variables. To compare the HADS anxiety and depression scores at three assessments according to age, sex and patient or spousal condition, we performed generalized estimating equations with a first-order autoregressive correlation matrix between assessments, assuming a normal marginal distribution and identity link function. To compare IES scores at 30 and 90 days, we performed generalized estimating equations with a first-order autoregressive correlation matrix between assessments, assuming a Poisson marginal distribution and log link function. Generalized estimating equations are a powerful and modern methods for repeated measures, allowing the fit of continuous, categorical and count outcomes. Based on the literature, there was an expected natural trend to improvement over time in HADS and IES. To be correctly modelled, we used an autoregressive correlation matrix, assuming that the measures that were taken closer were more correlated than were those taken over far distances. Therefore, due to their flexibility and features, allowing correct model specification and taking account of all measures, we used generalized estimating equations instead of ANOVA.^(14)^

We tested interactions among sex, condition and time of assessment. Multiple comparisons were adjusted for multiplicity using Bonferroni's method. A p value of < 0.05 was considered significant.

## RESULTS

From March 2011 to March 2013, 1125 patients were admitted to the ICU ≥ 48 hours. After the inclusion and exclusion criteria were applied (588 exclusion criteria and 154 refused to participate), we analyzed 118 patients and their spouses.

Ninety-six (81.4%) patients were male. The mean age was 58.8 ± 12.6 years for patients and 54.8 ± 12.6 for spouses. The spouses were, on average, 4.0 years younger than the patients (95% confidence interval - 95%CI: 2.7 to 5.4 years; p < 0.001). There was no significant difference in the proportion of patients and spouses aged over 60 years. Among spouses, we observed a significantly lower proportion with college education (p < 0.001) (Table 1).

**Table 1:** Characteristics of patients and their spouses

Variables	Values
Patients (n = 118)	
Age	58.8 ± 12 (30 - 84)
Sex (male)	96 (81.4)
College education	102 (86.4)
Moderate-severe pain at ICU	60 (50.8)
SOFA score	1.5 [0 - 5]
SAPS 3 (points)	46.5 [36 - 61]
Cancer	67 (56.8)
Activity of daily lives - eigh dependence	10 (8.5)
Vasopressors	43 (36.4)
Mechanical ventilation > 24 hours	28 (23.7)
Renal replacement therapy	19 (16.1)
Clinical ICU admission	66 (55.9)
ICU length of stay (days)	5.63 ± 5.92 (2 - 47)
Spouses (n = 118)	
Age	54.8 ± 12 (24 - 81)
Sex (male)	22 (18.6)
College education	84 (71.2)
Catholic religion	86 (72.9)
No previous family experience with ICU	28 (23.7)
Family staying time in ICU (hours/day)	16.00 [12 - 24]
ICU satisfaction score (points)	13 [12 - 14]

ICU - intensive care unit; SOFA - Sequential Organ Failure Assessment; SAPS - Simplified Acute Physiology Score. The results are expressed as mean ± standard deviation, n (%) or median [IQR].

We observed that 60 (50.8%) patients had severe pain during the ICU stay, 67 (56.8%) had a cancer diagnosis and 66 (55.9%) were receiving clinical treatments (Table 1). During the ICU stay, we observed that ten (8.5%) patients were very dependent in terms of their activities of daily living, 28 (23.7%) needed mechanical ventilation, 43 (36.4%) used vasoactive drugs and 19 (16.1%) needed renal replacement therapy. The median SOFA score was 1.5, and the median SAPS 3 score was 46.5 points, with a range of 21 - 93 (Table 1).

After 30 days, we followed-up 88 (74.6%) patients and 103 (87.3%) spouses, and at 90 days, we followed-up 77 (65.2%) patients and 94 (79.7%) spouses. The reasons for missing data were deaths (11 at 30 days and 4 at 90 days); being in no condition to participate (seven patients at 30 and 90 days); two patients refusing to participate at 30 days; and lost to follow-up (10 at 30 days and nine at 90 days).

The scores of anxiety, depression and PTSD symptoms are displayed in table 2. We observed that both patients and their spouses had higher anxiety- and depression-HADS scores as well as total HADS scores at baseline during their ICU stay compared with the scores after 30 and 90 days. Twenty-two (18.6%; 95%CI: 12.1% to 26.9%) patients had symptoms of anxiety, and eight (6.8%; 95% CI: 3.0% to 12.9%) had symptoms of depression at baseline. After 30 and 90 days, no patients had symptoms of anxiety, and few patients had symptoms of depression (1.1% [95%CI: 0.3% to 6.2%] and 1.3% [95%CI: 0.3% to 7.0%], respectively).

**Table 2:** Anxiety, depression and post-traumatic stress disorder symptoms among patients and their spouses

	HADS total Score*	HADS Subscale Anxiety score*	HADS Subscale Depression score*	IES score†
Patients				
At ICU (n = 118)	9.9 ± 7.2	6.7 ± 4.4	3.2 ± 3.6	-
30-day (n = 88)	3.4 ± 4.6	1.8 ± 2.3	1.6 ± 2.6	0.0 [0.0 - 6.0]
90-day (n = 77)	1.9 ± 3.7	1.0 ± 1.8	0.9 ± 2.2	0.0 [0.0 - 0.0]
Spouses				
At ICU (n = 118)	10.8 ± 7.2	7.7 ± 4.4	3.1 ± 3.7	
30-day (n = 103)	7.1 ± 7.8	4.1 ± 4.2	3.1 ± 4.1	5.0 [0.0 - 10.0]
90-day (n = 94)	5.4 ± 7.6	3.1 ± 4.1	2.2 ± 3.8	0.0 [0.0 - 1.0]

HADS - Hospital Anxiety and Depression Scale; IES - Impact of Event Scale; ICU - intensive care unit.

* Mean ± standard deviation;

† Median [CI].

Among the spouses, twenty-seven (22.9%; 95%CI: 15.6% to 31.5%) had symptoms of anxiety, and eight (6.8%; 95%CI: 3.0% to 12.9%) had symptoms of depression at baseline. After 30 days, 11 (10.7%; 95%CI: 5.4% to 18.3%) had symptoms of anxiety and six (5.8%; 95%CI: 2.2% to 12.2%) had symptoms of depression. These symptoms decreased after 90 days: seven (7.4%; 95%CI: 3.0% to 14.7%) had symptoms of anxiety, and five (5.3%; 95%CI: 1,7% to 12.0%) had symptoms of depression.

**Figure 1:**
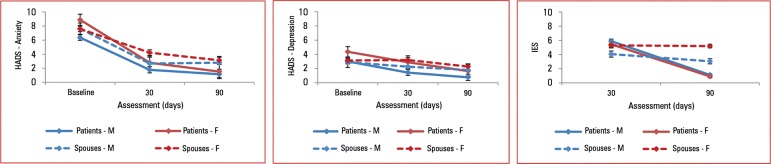
Hospital Anxiety and Depression Scale anxiety and depression subscales and Impact of Event Scale scores of posttraumatic stress disorder symptoms according to gender and spouse condition. HADS - Hospital Anxiety and Depression Scale; IES - Impact of Event Scale.

Overall, female sex was associated with higher scores, than were males in the anxiety HADS subscale, after adjusting for age, time of evaluation and whether they were patients or spouses (average +1.00 point [95%CI: 0.09 to 1.91]; p = 0.03). There was no significant difference between patients and spouses for anxiety (p = 0.098). We also observed that for each year older, both patients and spouses had an average reduction of 0.05 points (95%CI: -0.08 to -0.03; p < 0.001) on the HADS score for anxiety (Table 3). On average, these symptoms decreased over time, without an interaction of sex and condition. The score of anxiety was 4.23 (95%CI: -4.86 to -3.61; p < 0.001) and 5.05 (95%CI: -5.76 to -4.33; p < 0.001) points lower in the evaluation performed 30 and 90 days after ICU compared to baseline, respectively (Table 3). For depression, female sex was the only variable that was statistically associated with high scores (0.96 [95%CI: 0.07 to 1.85]; p = 0.034), whereas the level of depression decreased over time (Table 3). The outputs from the models for the HADS scores are depicted in figure 1.

**Table 3:** Generalized estimating equations with first order autoregressive correlation matrix comparing Hospital Anxiety and Depression Scale anxiety and depression and Impact of Event Scale scores over time, according to patient or spouse condition, gender and age

Parameters	Anxiety	Depression	PTSD
	Coefficient ± SD [95%CI]; p value	Coefficient ± SD [95%CI]; p value	Coefficient ± SD [95%CI]; p value
Female *versus* male	1.00 ± 0.46, [0.09 - 1.91]; 0.032	0.96 ± 0.45, [ 0.07 - 1.85]; 0.034	-0.08 ± 0.11, [ -0.29 - 0.13]; 0.472
Spouse *versus* patient	0.77 ± 0.46, [- 0.14 - 1.68]; 0.098	-0.04 ± 0.45, [-0.93 - 0.85]; 0.930	-0.37 ± 0.12, [-0.60 - -0.14]; 0.001
Age (per unit increase)	- 0.05 ± 0.01, [-0.08 - -0.03]; < 0.001	-0.02 ± 0.01, [-0.05 - 0.00]; 0.079	-0.04 ± 0.00, [ -0.04 - -0.03]; < 0.001
30-days *versus* baseline	-4.23 ± 0.32, [-4.86 - -3.61]; < 0.001	-1.44 ± 0.32, [ -2.07 - -0.80]; 0.001	-
90-days *versus* baseline*	-5.05 ± 0.37, [-5.76 - -4.33]; < 0.001	-0.67 ± 0.27, [ -1.20 - -0.14]; 0.013	-1.66 ± 0.19, [ -1.88 - -1.45]; < 0.001

PTSD - posttraumatic stress disorder; SD - standard deviation; 95%CI - 95% confidence interval.

* For posttraumatic stress disorder, the reference was the assessment at 30 days.

There was no association between sex and PTSD symptoms. However, spouses had higher IES scores than patients did (Table 3). Additionally, we found an interaction among sex, condition and time of assessment (Figure 1). Increasing age and later time of assessment were also associated with lower IES scores (Table 3).

## DISCUSSION

This study aimed to evaluate the association between sex and spouse conditions for symptoms of anxiety, depression and posttraumatic stress traumatic during ICU admission and at the 30- and 90-day follow-ups after ICU discharge. We observed that females had, on average, higher levels of anxiety and depression than males did, but there was no association with PTSD. By contrast, being a spouse or patient had no association with anxiety and depression, although spouses were associated with PTSD symptoms.

Several studies have demonstrated a high prevalence of anxiety and depression in critical ill patients and their family members. However, few data can address the sex differences regarding these conditions. Our results are in accordance with recent studies, where female sex was associated with symptoms of anxiety, depression and post-traumatic distress.^(4-9,11)^ In addition, their closest relatives suffered more from symptoms of post-traumatic stress, occasionally more so than the patients did.^(8,15)^ Research on the causes of psychological distress in females in midlife has focused on the current adversity and hormonal changes associated with menopause.^(16)^ However, psychosocial and unmeasured factors should also have an impact. We thus emphasize the need for the proper monitoring of anxiety, depression and PTSD, with greater attention paid to females, who are facing a critical illness during an ICU stay.

Posttraumatic stress occurs when a person experienced, witnessed, or was confronted with an event involving death or threat. It involves fear, helplessness or even intense terror. The ICU environment is so threatening that the prevalence of these symptoms was compared to that felt by people who experienced a natural disaster or even rape.^(9)^ We found that spouses were more vulnerable to post-traumatic stress symptoms than patients were. Interestingly, these symptoms in patients lessened significantly after three months, whereas they persisted over time in their spouses. However, although spouses spent many hours per day visiting the patients, we did not find an association between being a spouse and both anxiety and depression. Interestingly, the correlation of anxiety and depression symptoms between patients and family members in a positive direction was observed in a previous study by our group.^(8)^ In addition, anxiety, depression and post-traumatic stress symptoms in patients lessened significantly after three months, whereas in family members, they may persist over time.^(8)^

Although psychological distresses have been given considerable attention in recent years, few studies have addressed the symptoms of anxiety, depression and post-traumatic distress by comparing patients and their spouses or exploring the effects of sex and spouse conditions, including the ICU stay and 30 and 90 days after ICU discharge.^(17,18)^

Our study has some strengths, such as the assessment of patients and their respective spouses at three time points (during the ICU stay and at 30 and 90 days after ICU discharge) and the evaluation of exact patient-spouse pairs. However, it has some limitations. First, it was a single-center study that was performed in an open-visit private ICU; therefore, it was not representative of Brazilian ICUs.^(19)^ Second, we excluded several patients (mainly due to severity and barriers to completing the interview), although we could analyze a considerable number of pairs (patient/spouse). Third, we had a very low incidence of anxiety and depression symptoms compared with the literature. Part of this could be attributed to our open-visit policy, but we cannot rule out selection bias by excluding more severe patients.

## CONCLUSIONS

In our study setting, we found that female sex had an important association with anxiety and depression, whereas spouses were associated with post-traumatic stress symptoms, which persisted over time. We recommend a proactive intervention during intensive care unit stays to reduce patient-spouse distresses.

### Authors' contributions

Conception or design of the work: RR Fumis and HS Oliveira; Acquisition of data: RR Fumis; Statistical Analysis: RR Fumis; Interpretation of data: RR Fumis and HS Oliveira; Drafting the manuscript: RR Fumis and HS Oliveira; Final approval of the version to be published: RR Fumis and HS Oliveira.
